# Exposure to incense burning, biomarkers, and the physical health of temple workers in Taiwan

**DOI:** 10.1007/s11356-023-29420-w

**Published:** 2023-09-02

**Authors:** Su-Er Guo, Pamela A. Ratner, Sung-Chih Tseng, Chieh-Mo Lin, Miao-Ching Chi, Chiang-Wen Lee, Ying-Chun Yu

**Affiliations:** 1 Department of Nursing and Graduate Institute of Nursing, College of Nursing, Chang Gung University of Science and Technology (CGUST), Chiayi County, 613016 Taiwan; 2 Chronic Diseases and Health Promotion Research Center, CGUST, Chiayi County, 613016 Taiwan; 3 Department of Neurology, Chang Gung Memorial Hospital (CGMH) Chiayi Branch, Chiayi County, 613 Taiwan; 4 Department of Safety Health and Environmental Engineering, Ming Chi University of Technology, New Taipei City, 24301 Taiwan; 5 School of Nursing, University of British Columbia, Vancouver, BC V6T 2B5 Canada; 6 Family Medicine Department, CGMH Chiayi Branch, Chiayi County, 61363 Taiwan; 7 Division of Pulmonary and Critical Care Medicine, CGMH Chiayi Branch, Chiayi County, 61363 Taiwan; 8 Graduate Institute of Clinical Medical Sciences, College of Medicine, Chang Gung University (CGU), Taoyuan City, 33302 Taiwan; 9 Department of Respiratory Care, CGUST, Chiayi County, 613016 Taiwan; 10 Division of Basic Medical Sciences, CGMH Chiayi Branch, Chiayi County, 61363 Taiwan; 11 Department of Orthopaedic Surgery, CGMH Chiayi Branch, Chiayi County, 61363 Taiwan; 12 College of Medicine, CGU, Taoyuan City, 33302 Taiwan

**Keywords:** Incense burning, Particulate matter, Health status, Inflammatory cytokines, Lung function

## Abstract

Incense burning releases heavy particulate matter (PM) and nitrogen dioxide (NO_2_), known to have adverse effects on human health. Long-term exposure to PM and NO_2_ increases inflammatory cytokine levels and can induce respiratory diseases. This study examined the association between incense burning exposure and the health status, especially inflammatory biomarkers, of temple workers and volunteers in Taiwan. The longitudinal observational study compared adult temple workers and volunteers, with long-term incense burning exposure, to residents from outpatient clinics in the Chiayi area. Forced expiratory volume in 1 s (FEV1) and serum and exhaled breath condensate (EBC) cytokines were assessed. Nonparametric Mann-Whitney *U* tests were used to compare cytokine levels of the exposure and control groups during the cold and hot weather seasons. FEV1 was significantly more diminished in the exposed group than in the control group during the cold season. Exposure status was associated with greater hot-cold seasonal differences in serum interleukins (IL)-1β (regression coefficient (*B*) = 6.6, 95% confidence interval (CI) = 5.0 to 8.3, *p* < .001), IL17-A (*B* = 2.4, 95% CI = 0.3 to 4.5, *p* = .03), and plasminogen activator inhibitor [PAI]-1 (*B* = 5.4, 95% CI = 1.5 to 9.3, *p* = .009). After adjusting for confounders, the groups’ serum levels of IL-1β, IL-17A, and PAI-1 significantly differed. EBC cytokines did not show significant differences. Elevated levels of IL-1β, IL17-A, and PAI-1 have been associated with various autoinflammatory syndromes and diseases. Given the cultural significance of incense burning, culturally sensitive interventions, including education, policy development, and program implementation, are crucial to protect individuals’ health, especially temple workers, from the adverse effects of exposure, addressing the manufacture, importation, and sale of incense.

## Introduction

The burning of incense, a long-standing ritual in temples and homes for worship purposes, releases heavy air pollutants, such as particulate matter (PM_10_ and PM_2.5_) (Lin et al. 2008; Kuo et al. 2016), polycyclic aromatic hydrocarbons (PAHs), nitrogen dioxide (NO_2_), and other carcinogens, posing risks to human health (Lin et al. 2007; Liou et al. 2008; Yang et al. 2012; Shen et al. 2017). Studies in Taiwan have shown that burning incense can lead to high concentrations of PM_2.5_ (185–625 μg/m^3^) during certain events and inside temples, far exceeding recommended levels (Bootdee et al., 2016). In Taiwan, PM_10_ concentrations inside two temples (539 μg/m^3^ and 626 μg/m^3^, respectively) were 4 to 6 times those outside the temples (gates 148 μg/m^3^, street 121 μg/m^3^), and visitors were exposed to about 512 μg/m^3^ in the temples during worshipping dates (Lung and Kao 2003). Another study also indicated that PM_1_ concentrations (120.3 ± 48.8 μg/m^3^) and PM_2.5_ concentrations (149.9 ± 59.6 μg/m^3^) on event days were higher than those non-event days (PM_1_, 62.8 ± 47.9 μg/m^3^; PM_2.5_, 83.4 ± 59.6 μg/m^3^) in Taiwan (Hien et al. 2022). In addition, according to our ongoing investigation (unpublished), the indoor data of the same temple showed that PM_2.5_ concentrations exceed Taiwan standard by 5–18 times (401.44±220.46 μg/m^3^). Moreover, PAHs released from incense burning have also been measured at significant levels, with potential implications for cancer risk (Kuo et al. 2003; Chiang and Liao 2006; Chen et al. 2022). Therefore, long-term exposure to these carcinogens from incense smoke can have adverse effects on human health, particularly on the respiratory system and brain connectivity (Lin et al. 2008; Chen et al. 2017; Wong et al. 2020).

Smokers exposed to indoor incense burning, in addition to cigarette smoke, have been found to have an increased risk of lung cancer; high levels of cumulative incense exposure and cigarette smoking seem to have a synergistic effect (Tse et al. 2011). Chen et al. (2022) subsequently found that indoor incense burning, even in the absence of cigarette smoking, is associated with an increased risk of lung cancer. It is capable of initiating oxidative stress (cell and tissue damage), leading to systemic or chronic inflammation and pulmonary diseases such as asthma and chronic obstructive pulmonary disease (COPD), as well as sepsis-induced acute lung injury (MacNee 2001; Caramori and Papi 2004; Guo and Ward 2007; Hoshino and Mishima 2008; Pizzino et al. 2017). Polycyclic aromatic hydrocarbons (PAH), found in incense smoke, can also damage cells and cause proinflammatory effects (Miousse et al. 2015; Tong et al. 2019).

Human health can be adversely affected by both incense burning and outdoor air pollution. In Taiwan, rituals involving burning incense are conducted indoors in semi-open or closed temples, leading to higher air pollution levels (Hien et al. 2022). Similarly, a study in Hong Kong found that temples located in urban and residential areas, whether semi-open or closed, could impact the health of nearby pedestrians and residents (Cai and Wong 2021). Such rituals can elevate air pollutant concentrations around the temples beyond background levels, influencing the health of temple workers, worshippers, and residents (Lau and Luk 2001; Cai and Wong 2021).

Moreover, atmospheric pollution exhibits seasonal variation, with fine particulate matter (PM_2.5_) showing higher daily mean concentrations during cold weather seasons compared to warm seasons (Hwang et al. 2018). In Taiwan, air pollution worsens in the cold season, and higher PM_2.5_ levels are associated with increased hospital emergency room visits for respiratory diseases (Hwang et al. 2017). Analyzing data from the Beijing Environmental Center in 2016, Lu et al. (2018) observed worse PM_2.5_, NO_2_, and carbon monoxide (CO) pollution in autumn and winter than in spring and summer, while PM_10_ pollution was worse in spring and autumn than in winter and summer.

To prevent the occurrence of disease or to ensure early diagnosis, detection of the short-term immediate impacts of air pollution is critical. Short-term exposure to PM_2.5_ and NO_2_ can cause a neutrophil-mediated airway inflammatory response, which can lead to clinical symptoms (Guo et al. 2020a, b, c). Early detection can motivate medical therapy or the reduction of exposure to major pollutants and effectively minimize any clinical symptoms. Therefore, we conjectured that detecting inflammatory responses could be a rapid and direct method of understanding the impact of burning incense on human health (Di et al. 2021).

Studies of the effects of air pollution on inflammation have mostly focused on serum analyses of fibrinogen, interleukin (IL)-1β, IL-6, IL-17, PAI-1, 8-hydroxy-2-deoxyguanosine (8-OHdG)-tumor necrosis factor (TNF)-alpha, and high-sensitivity C-reactive protein (hs-CRP). However, collecting exhaled breath condensate (EBC) may also be effective in evaluating the effects of air pollution on inflammatory responses (Seifi et al. 2021). EBC, a non-invasive method of collecting airway lining fluid, is a simple way to observe the pulmonary environment (Pirozzi et al. 2015). It is a promising method to reflect the levels of inflammatory biomarkers and monocyte chemoattractant protein (MCP)-1 and to identify respiratory or pulmonary-related diseases (Kharitonov and Barnes 2001; Montuschi and Barnes 2002; Fireman et al. 2019).

Exposure to burning incense can increase inflammation. Many studies have focused on the effects of incense burning on serum components (Fang et al. 2003; Pandey et al. 2013); few studies have examined the impact of incense burning on inflammatory cytokines (Navasumrit et al. 2008). Hence, the association between incense burning and systemic inflammatory cytokines needs to be studied further. Inflammatory responses are the most direct effect of burning incense exposure on health. We investigated the effects of burning incense on inflammatory biomarkers among temple workers and other residents in the Chiayi area, Taiwan.

## Material and methods

### Study design and participants

A longitudinal observational design was employed. Participants were divided into two groups: exposed and control. The former group included workers and volunteers, recruited from a temple in Chiayi County, Taiwan, who had long-term exposure to burning incense. The control group included residents of the Chiayi area recruited from outpatient clinics of a regional hospital’s family medicine department. The inclusion criteria for the participants were (1) lived in the Chiayi area for at least 1 year; (2) aged 18 years or older; and (3) had the ability to understand and communicate in Mandarin or Taiwanese. Patients with any known diseases, such as heart disease, asthma, tuberculosis, autoimmune diseases, or cancer, which would have been associated with inflammation indistinguishable from PM-associated inflammation were excluded from the control group, as were patients who had undergone surgery within 1 month of enrollment. Age and sex were matched between the exposed and control groups. We scheduled two follow-up visits between November 2019 and December 2020. One follow-up visit was conducted in the hot weather season (May–November) and one in the cold season (December–April).

A power analysis, conducted to determine an adequate sample size for analyses of covariance with statistical power of .80, a medium effect size of .25, and an alpha level of 0.05 (Guo et al. 2020a, b, c), showed that a target sample size of 128 participants was sufficient. Thus, the targeted total sample size was 64 participants per group. Ethical approval was obtained from the Institutional Review Board (IRB) of the Chang Gung Medical Foundation (IRB No. 201901540B0C501, approved on October 28, 2019).

**Fig. 1:**
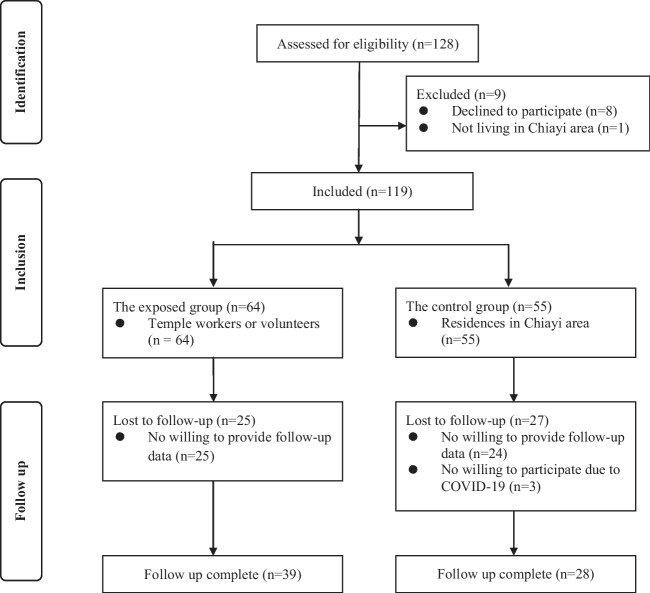
Study enrollment flowchart

Participants, who are temple workers or volunteers, in the exposed group (*n* = 64) were recruited before the control group participants were recruited. Sixty-four potential participants were asked to participate in the control group; however, eight of them declined to participate and one did not live in the Chiayi area. In total, 119 participants consented to participate. During the study, 25 participants in the exposed group and 27 in the control group were lost to follow-up because they did not complete the follow-up questionnaires or blood tests; additionally, two participants withdrew because of concerns about the COVID-19 pandemic and were excluded from the final analysis. Consequently, the data of 67 participants were included in the final analysis, 39 in the exposed group and 28 in the control group (see Fig. 1 for details).

### Demographic and health characteristics of participants

After explaining the objectives and procedures of the study, informed consent was obtained from those who were willing to participate. We requested enrolled participants to complete a questionnaire through an interview, to provide blood samples, and to undergo a lung function test. The questionnaire obtained information about the participants’ sex, age, occupation, religion, marital status, education level, and diagnoses, so that a Charlson Comorbidity Index could be calculated. The Charlson Comorbidity Index is a method of categorizing comorbidities of patients based on the International Classification of Diseases (ICD) and was first developed by Charlson et al. as a weighted index to predict risk of death within 1 year of hospitalization for patients with specific comorbid conditions. The environmental conditions of the participants’ private residences and lifestyles (i.e., exercise, smoking history, environmental tobacco smoke exposure, incense burning, and diet) were also collected so that potential confounding variables could be controlled. All of the participants were required to complete the questionnaire items during each follow-up.

### Air quality

To investigate the impact of exposure to burning incense on inflammatory biomarkers in serum and EBC, PM_2.5_ and NO_2_ data were acquired from official monitoring stations of the Taiwan Air Quality Monitoring Network (TAQMN). To measure the exposure to burning incense, PM_2.5_ and NO_2_ concentrations were adopted from the 24-h average data acquired from the stations near the temple or private residences where the participants were located. Data from a monitoring station near these areas were considered appropriate to evaluate personal exposure (Di et al. 2021).

### Inflammatory biomarkers

#### Exhaled breath condensate (EBC)

EBC was collected as a liquid on an Eco screen using a Turbo DECCS (Medivac, Parma, Italy) within 10–15 min and stored at −80 °C. EBC examination is a non-invasive and safe method of collecting airway lining fluid to observe the pulmonary environment (Seifi et al. 2021; Pirozzi et al. 2015), and is used to evaluate pulmonary inflammation. It is a promising method to reflect the levels of inflammatory biomarkers (such as IL-1β, IL-6, and IL-8) and identify respiratory or pulmonary diseases (Kharitonov et al. 2001; Montuschi et al. 2002; Fireman et al. 2019). In the current study, EBC was collected to analyze the levels of inflammatory cytokines, including IL-1β, IL-6, IL-8, IL-17A, and MCP-1. The inflammation levels of these cytokines were determined using an enzyme-linked immunosorbent assay (ELISA) kit using ELISA MAX™ Deluxe Set (BioLegend, San Diego, CA, USA). Absorbance at 450 and 540 nm was measured using an ELISA reader.

#### Blood biomarkers

Blood samples were collected to determine the serum concentration of inflammatory cytokines, including IL-1β, IL-6, IL-8 IL-17A, TNF-α, hs-CRP, a biomarker of oxidative stress, 8-OHdG, and inflammatory responses of participants. The 8-OHdG level is also an indicator of PAHs. The serum levels of IL-1β, IL-6, IL-8, IL-17A, and TNF-α were determined with an ELISA kit using ELISA MAX™ Deluxe Set (BioLegend, San Diego, CA, USA). The hs-CRP levels were measured with an ELISA kit using a Human C-Reactive Protein ELISA Kit (Assaypro, St Charles, MO, USA). ELISA was performed to determine PAI-1 concentrations using the Human PAI-1 Assay Max ELISA Kit (Assaypro, St Charles, MO, USA). 8-OHdG levels were measured using an 8-OHdG ELISA Kit (Finetest, Wuhan, Hubei, China). Absorbance at 450 and 540 nm was measured using an ELISA reader.

### Dyspnea and lung function

The modified Medical Research Council (mMRC) dyspnea scale was used to assess dyspnea among participants. The mMRC scale ranges from 0 to 4, with a higher value indicating a more severe state of dyspnea; the scale has been shown to have good reliability (Tsai et al. 2012). Each study participant underwent a lung function test in both the hot and cold weather seasons. A spirometer (BTL-08 Spiro Pro; BTL, UK) was used to measure the forced expiratory volume in 1 s (FEV_1_), forced vital capacity (FVC), and the FEV_1_/FVC.

### Self-care knowledge of particulate matter

Self-care knowledge of particulate matter (PM) was a questionnaire developed by the research team and consisted of 16 items about self-care knowledge regarding air pollution (Guo et al. 2020a, b, c). The results were evaluated by three experts for scale-content validity, which was found to be .92, thus, exhibiting good validity, based on a study by Shi (2012). Additionally, Cronbach’s alphas of the original and current studies were .84 and .81, respectively. Participants with higher scores demonstrated superior self-care knowledge regarding air pollution.

### Statistics

Demographics and characteristics of the exposed and control groups were compared using independent *t*-tests for continuous variables and Fisher’s exact tests for categorical variables. The mMRC scores were compared using the Mann-Whitney *U*-test due to non-normal distribution. Wilcoxon signed-rank test was used to compare changes in serum tests and EBC between cold and warm seasons (combined exposed and control groups). Differences in serum tests and EBC between exposed and control group participants were assessed using the Mann-Whitney *U*-test. Quantile regression was employed to evaluate the association between exposure status and weather changes (hot-cold difference) in serum tests/EBCs, while controlling for confounding factors. Covariates included waist-to-hip ratio, education level, self-care knowledge of PM, smoking by family members in the house, exposure to burning incense, PM_2.5_, and NO_2_ in the living township and some health behaviors (use of air purifiers, and total duration of exercise). The desired quantile was the 50^th^ percentile and the standard error was calculated under the assumption of independent and identically distributed errors. Statistical analyses were conducted using SPSS 26 software (IBM SPSS, Chicago, IL, USA). SPSS 26 software was used for statistical analyses, with a significance level set at *p* < .05.

## Results

### Demographics and characteristics

Female participants outnumbered male participants (40 (59.7%) vs. 27 (40.3%)). The mean age, in the cold weather season, was 61.2 years (standard deviation (SD) = 10.9 years), ranging from 33 to 82 years. Table 1 presents the demographics and characteristics of the exposed and control group participants. The exposed group participants were more likely to have religious beliefs, lower education levels, and poorer self-care knowledge of PM, and were more likely to be exposed to burning incense than the control group participants, in both the cold and hot weather seasons. The lung symptoms (mMRC score and FEV_1_) were significantly more severe in the exposed group than in the control group in the cold weather season but not in the hot season. Furthermore, ambient PM_2.5_ and NO_2_ levels were significantly lower in the exposed group during the cold season, whereas they were significantly higher in the exposed group during the hot season (see Table 1).

**Table 1:** Demographics and characteristics of the exposed and control participants in the cold and warm seasons

Variable	Cold season			Warm season		
	Exposed (*n* = 39)	Control (*n* = 28)	*p*	Exposed (*n* = 39)	Control (*n* = 28)	*p*
Age, year	62.4 ± 10.6	59.6 ± 11.2	0.296	63.3 ± 10.6	59.9 ± 11.3	0.206
Male sex	15 (38.5)	12 (42.9)	0.803	15 (38.5)	12 (42.9)	0.803
Body mass index, kg/m^2^	25.5 ± 3.8	25.3 ± 3.8	0.848	25.7 ± 3.8	25.1 ± 3.9	0.522
Waist to hip ratio (*n* = 133)	0.92 ± 0.06	0.89 ± 0.07	0.093	0.91 ± 0.06	0.89 ± 0.07	0.395
Living arrangement			0.285			0.285
Living alone	7 (17.9)	2 (7.1)		7 (17.9)	2 (7.1)	
Living with others	32 (82.1)	26 (92.9)		32 (82.1)	26 (92.9)	
Religion belief			0.010			0.010
No	0 (0.0)	5 (17.9)		0 (0.0)	5 (17.9)	
Yes	39 (100.0)	23 (82.1)		39 (100.0)	23 (82.1)	
Education level, year	8.5 ± 4.5	13.1 ± 4.7	<0.001	8.6 ± 4.2	13.1 ± 4.7	<0.001
Smoking habit			0.391			0.391
Never/quit	35 (89.7)	27 (96.4)		35 (89.7)	27 (96.4)	
Current smoker	4 (10.3)	1 (3.6)		4 (10.3)	1 (3.6)	
Lung symptoms						
mMRC score	1.0 [0.0, 1.0]	0.0 [0.0, 1.0]	0.005	0.0 [0.0, 1.0]	0.0 [0.0, 1.0]	0.378
FEV_1_, %	70.7 ± 26.8	87.0 ± 19.4	0.008	74.5 ± 21.3	78.4 ± 26.8	0.572
Pulmonary function			0.057			0.993
Mild	17 (43.6)	20 (71.4)		18 (46.2)	13 (46.4)	
Moderate	13 (33.3)	7 (25.0)		13 (33.3)	9 (32.1)	
Severe or extremely severe	9 (23.1)	1 (3.6)		8 (20.5)	6 (21.4)	
Skin allergy	13 (33.3)	10 (35.7)	1.000	14 (35.9)	10 (35.7)	1.000
Respiratory tract allergy	11 (28.2)	7 (25.0)	1.000	10 (25.6)	7 (25.0)	1.000
Knowledge for indoor air pollution	10.8 ± 2.3	12.8 ± 2.0	0.001	11.4 ± 2.0	13.1 ± 2.2	0.001
Use of air purifier	7 (17.9)	11 (39.3)	0.092	7 (17.9)	14 (50.0)	0.008
Use of range hood	37 (94.9)	28 (100.0)	0.506	36 (92.3)	28 (100.0)	0.259
Smoke of the family members in the house	11 (28.2)	2 (7.1)	0.057	11 (28.2)	3 (10.7)	0.128
The frequency of smoking for the primary smoked family member			0.047			0.130
No	28 (71.8)	26 (92.8)		28 (71.8)	25 (89.3)	
>1 pack per day	4 (10.3)	2 (7.1)		7 (17.9)	3 (10.7)	
≤1 pack per day	7 (17.9)	0 (0.0)		4 (10.3)	0 (0.0)	
Take a notice of air quality index	21 (53.8)	15 (53.6)	1.000	20 (51.3)	18 (64.3)	0.326
Burning incense at home			0.001			0.006
No	4 (10.3)	11 (39.3)		6 (15.4)	11 (39.3)	
Once in a while	8 (20.5)	10 (35.7)		10 (25.6)	11 (39.3)	
Everyday	27 (69.2)	7 (25.0)		23 (59.0)	6 (21.4)	
Total score of dietary habit scale	69.6 ± 7.0	70.3 ± 7.8	0.709	72.2 ± 7.4	72.6 ± 6.7	0.847
Exercise (per week)						
Total duration of exercise, min	28.7 ± 18.9	41.3 ± 32.4	0.051	29.5 ± 20.0	31.8 ± 27.3	0.690
Total duration of exercise ≥150 min	19 (48.7)	12 (42.9)	0.804	17 (43.6)	12 (42.9)	0.576
Charlson Comorbidity Index score	3.0 ± 1.2	2.8 ± 1.2	0.455	3.1 ± 1.3	2.8 ± 1.2	0.333
Fine particulate matter of 2.5 μm or less in diameter (PM_2.5_), μg/m^3^	19.3 ± 3.4	27.3 ± 10.8	<0.001	13.8 ± 8.5	10.0 ± 6.0	<0.001
Nitrogen dioxide (NO_2_), ppm	8.8 ± 1.2	10.1 ± 3.7	<0.001	8.2 ± 0.9	6.5 ± 2.9	<0.001

Data are presented as frequency (percentage), mean ± standard deviation, or median (25^th^ and 75^th^ percentiles)

*Abbreviations*: *FEV*_*1*_, forced expiratory volume in 1 s; *mMRC*, Modified Medical Research Council

Based on the study design, EBC samples were collected in the hot and cold weather seasons; however, some participants could only collect EBC once during the study period because of the social interruption caused by the COVID-19 pandemic. Therefore, we excluded the participants with only one EBC dataset (*n* = 16); consequently, 118 EBC datasets from the two groups were analyzed.

### Serum tests and EBC between the cold and warm seasons

We compared serum tests and EBC between the cold and warm seasons (see Table 2). The results showed significant changes in all these parameters (*p* < .05). Specifically, hs-CRP and 8-OHdG values decreased significantly from the cold to warm seasons, while the values of other parameters significantly increased during this period.

**Table 2:** Serum tests and exhaled breath condensates of participants between the cold and warm seasons, combining the exposed and control groups

Variable	Cold season	Warm season	*p*
Serum test			
IL-1β, pg/mL	0.6 [0.0, 2.6]	7.2 [3.5, 7.9]	<0.001
IL-6, pg/mL	2.9 [1.0, 7.7]	10.1 [6.4, 14.8]	<0.001
IL-8, pg/mL	5.0 [2.0, 11.5]	18.8 [13.9, 21.5]	<0.001
IL17-A, pg/mL	2.3 [0.3, 3.1]	9.0 [6.9, 10.5]	<0.001
TNF-α, pg/mL	2.3 [0.7, 3.3]	8.7 [4.5, 11.7]	<0.001
hsCRP, ng/mL	1635.4 [582.8, 3652.8]	1055.6 [377.6, 2120.8]	0.003
PAI-1, ng/mL	10.6 [9.0, 12.6]	20.4 [17.5, 23.1]	<0.001
8-OhdG, ng/mL	12.4 [9.4, 21.8]	10.3 [7.9, 17.8]	0.011
Exhaled breath condensate			
IL-1β, pg/mL	2.0 [1.1, 2.8]	2.3 [1.6, 3.4]	0.002
IL-6, pg/mL	5.7 [0.3, 6.6]	8.0 [7.4, 9.6]	<0.001
IL-8, pg/mL	0.0 [0.0, 0.3]	6.3 [5.0, 8.7]	<0.001
IL-17A, pg/mL	0.8 [0.2, 1.3]	2.9 [2.7, 3.4]	0.006
MCP-1, pg/mL	1.8 [1.2, 3.2]	4.5 [4.3, 4.8]	0.013

Data were presented as median (25^th^ and 75^th^ percentiles)

*Abbreviations*: *IL*, interleukin; *TNF-α*, tumor necrosis factor-alpha; *hs-CRP*, high-sensitivity C-reactive protein; *PAI-1*, plasminogen activator inhibitor-1; *8-OHdG*, 8-hydroxy-2-deoxyguanosine; *MCP-1*, monocyte chemoattractant protein-1

### Serum tests and EBC of the exposed and control group participants

Serum tests and EBC of the exposed and control group participants were compared in the cold and hot weather seasons (see Table 3). We found that the serum IL17-A and TNF-α levels were significantly higher in the exposed group than in the control group in both seasons. The serum IL-6 and hs-CRP levels were significantly higher in the exposed group than in the control group in the cold season, whereas the serum IL-1β and IL-8 levels were significantly higher in the exposed group in the hot season. The 8-OHdG level was significantly lower in the exposed group during the hot season. In terms of the EBC, the IL-6 and MCP-1 levels were significantly higher in the exposed group than in the control group during the cold weather season, but not during the hot season. The IL-8 levels were significantly lower in the exposed group during both seasons (see Table 3).

**Table 3:** Serum tests and exhaled breath condensates of the exposed and control participants in the cold and warm seasons

Variable	Cold season			Warm season		
	Exposed (*n* = 39)	Control (*n* = 28)	*p*	Exposed (*n* = 39)	Control (*n* = 28)	*p*
Serum test						
IL-1β, pg/mL	0.6 [0.2, 2.0]	0.4 [0.0, 4.1]	0.890	7.5 [7.0, 8.2]	1.0 [0.0, 7.5]	<0.001
IL-6, pg/mL	4.8 [2.7, 9.7]	0.9 [0.1, 2.6]	<0.001	10.5 [8.0, 14.2]	8.8 [2.6, 15.5]	0.101
IL-8, pg/mL	5.3 [3.5, 10.0]	2.1 [0.0, 12.7]	0.170	20.5 [18.0, 22.5]	15.2 [10.9, 19.3]	0.009
IL17-A, pg/mL	2.9 [2.3, 3.7]	0.3 [0.0, 0.6]	<0.001	10.0 [9.0, 11.7]	2.0 [0.4, 7.5]	<0.001
TNF-α, pg/mL	2.9 [2.3, 3.7]	0.7 [0.1, 1.4]	<0.001	9.7 [8.0, 12.3]	2.5 [0.9, 10.0]	0.001
hsCRP, ng/mL	2066 [822, 4516]	1264 [184, 2238]	0.032	1187 [588, 2087]	911 [273, 2212]	0.403
PAI-1, ng/mL	10.5 [9.2, 11.3]	15.2 [8.6, 29.1]	0.078	21.2 [18.3, 22.6]	18.8 [15.5, 23.7]	0.223
8-OhdG, ng/mL	12.3 [8.9, 19.9]	14.5 [10.2, 26.3]	0.233	8.9 [7.2, 14.5]	13.1 [8.8, 24.1]	0.035
Exhaled breath condensate						
IL-1β, pg/mL	1.9 [1.1, 2.4]	2.5 [0.8, 3.0]	0.469	2.3 [1.9, 3.4]	1.9 [1.1, 4.2]	0.369
IL-6, pg/mL	6.3 [5.9, 7.3]	0.0 [0.0, 0.7]	<0.001	7.6 [7.4, 9.6]	9.4 [8.6, 10.8]	0.121
IL-8, pg/mL	0.0 [0.0, 0.0]	11.9 [0.3, 23.1]	<0.001	5.3 [4.7, 7.3]	8.3 [7.3, 11.3]	0.002
IL-17A, pg/mL	0.7 [0.3, 2.4]	0.8 [0.0, 1.1]	0.110	3.0 [2.8, 3.4]	2.8 [2.5, 3.6]	0.223
MCP-1, pg/mL	3.0 [1.3, 5.2]	1.5 [0.8, 1.8]	<0.001	4.5 [4.0, 4.8]	4.5 [4.3, 5.3]	0.457

Data were presented as median (25^th^ and 75^th^ percentiles)

*Abbreviations*: *IL*, interleukin; *TNF-α*, tumor necrosis factor-alpha; *hs-CRP*, high-sensitivity C-reactive protein; *PAI-1*, plasminogen activator inhibitor-1; *8-OHdG*, 8-hydroxy-2-deoxyguanosine; *MCP-1*, monocyte chemoattractant protein-1

### Hot-cold differences in the exposed and control group participants

**Fig. 2:**
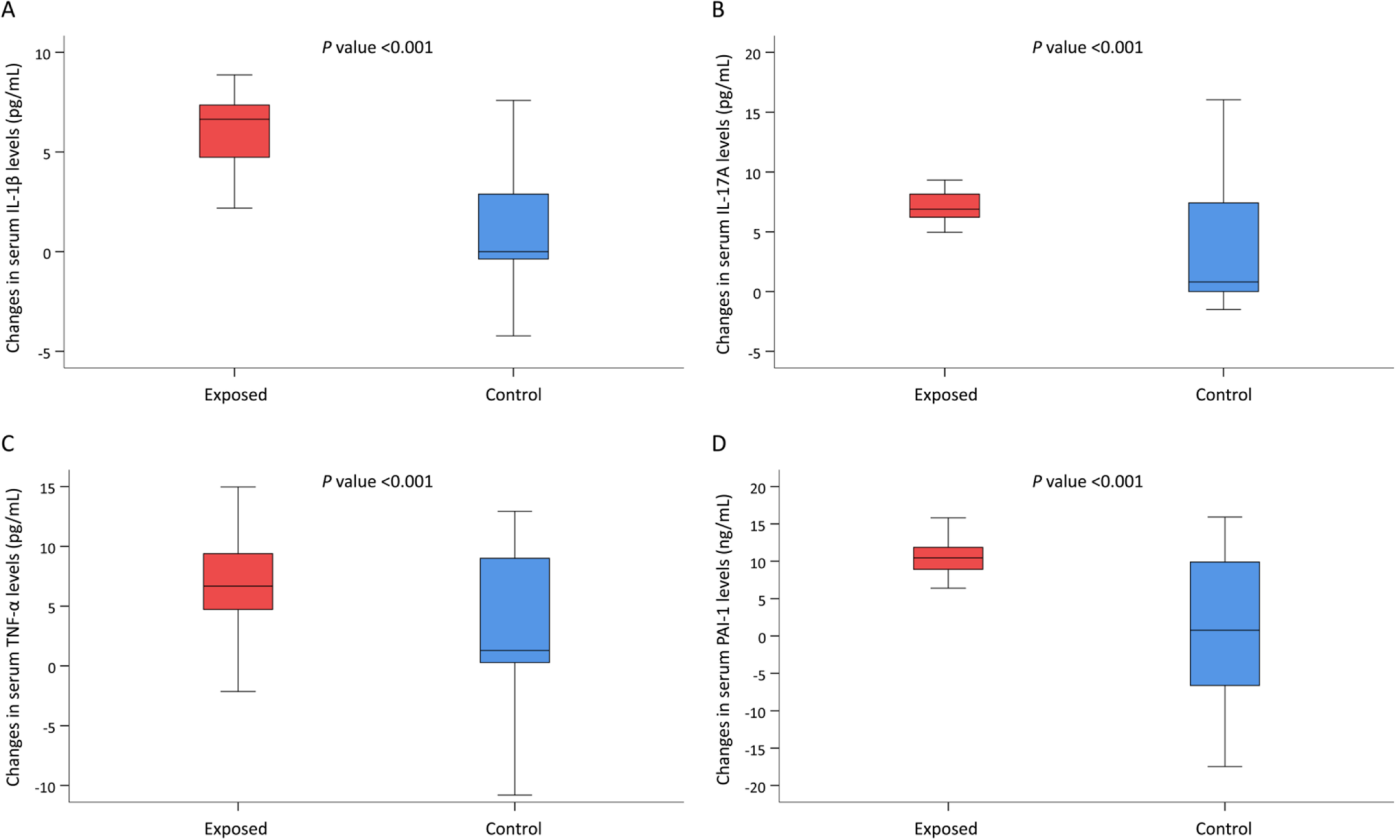
Distribution of serum IL-1β, IL17-A, TNF-α, and PAI-1 between the exposed and control groups

Table 4 shows the hot-cold weather differences between the exposed and control participants. The unadjusted quantile regression models demonstrated that the hot-cold differences in serum IL-1β, IL-6, IL-8, IL17-A, TNF-α, and PAI-1 were significantly greater in the exposed group than in the control group (all *p* values < .05; see the exact *p* values in Table 4). After multivariable adjustment, exposure status was significantly associated with a greater hot-cold difference in serum IL-1β (regression coefficient (*B*) = 6.6, 95% confidence interval (CI) = 5.0–8.3, *p* < .001), IL17-A (*B* = 2.4, 95% CI = 0.3–4.5, *p* = .03), and PAI-1 (*B* = 5.4, 95% CI = 1.5–9.3, *p* = .01). The exposed group had a greater hot-cold difference in serum TNF-α than the control group (*p* = .06). Figure 2 illustrates the distribution of serum levels of IL-1β, IL17-A, TNF-α, and PAI-1 in the two groups. In terms of EBC, the unadjusted quantile regression models showed that the hot-cold differences in IL-1β, IL-6, and IL-8 were significantly lower in the exposed group than in the control group (all *p* values < .05). However, exposure status was not independently associated with the hot-cold differences in all the EBC measurements, after adjusting for the covariates (see Table 4).

**Table 4:** Changes (warm season–cold season) in the serum tests and exhaled breath condensates of the exposed and control participants

Variable	Exposed	Control	Univariate analysis†		Multivariable analysis‡	
	(*n* = 39)	(*n* = 28)	*B* (95% CI)	*p*	*B* (95% CI)	*p*
Serum test						
IL-1β, pg/mL	6.7 [4.7, 7.4]	0.0 [−0.4, 2.9]	6.6 (5.6, 7.5)	<0.001	6.6 (5.0, 8.3)	<0.001
IL-6, pg/mL	5.4 [0.3, 9.1]	2.1 [0.1, 11.6]	3.2 (0.20, 6.2)	0.041	1.9 (−3.7, 7.5)	0.507
IL-8, pg/mL	14.3 [10.0, 16.0]	3.2 [0.2, 19.3]	10.3 (6.3, 14.2)	<0.001	2.7 (−4.5, 10.0)	0.463
IL17-A, pg/mL	6.9 [6.2, 8.2]	0.8 [0.0, 7.4]	5.4 (4.1, 6.7)	<0.001	2.4 (0.3, 4.5)	0.030
TNF-α, pg/mL	6.7 [4.7, 9.4]	1.3 [0.3, 9.0]	5.0 (2.5, 7.5)	<0.001	3.0 (−0.01, 6.1)	0.055
hs-CRP, ng/mL	−626 [−2841, 107]	70 [−842, 477]	−482 (−1049, 85)	0.101	−546 (−1418, 325)	0.225
PAI-1, ng/mL	10.5 [8.9, 11.9]	0.8 [−6.6, 9.9]	9.4 (7.1, 11.7)	<0.001	5.4 (1.5, 9.3)	0.009
8-OHdG, ng/mL	−2.5 [−5.7, 1.3]	−0.6 [−5.9, 2.8]	−1.7 (−4.9, 1.4)	0.290	0.54 (−3.4, 4.5)	0.787
Exhaled breath condensate						
IL-1β, pg/mL	0.4 [−0.1, 2.0]	2.1 [0.0, 3.0]	−1.5 (−2.6, −0.4)	0.010	−0.60 (−4.4, 3.2)	0.760
IL-6, pg/mL	1.7 [0.3, 3.5]	8.7 [4.3, 10.5]	−6.8 (−8.8, −4.8)	<0.001	−3.5 (−8.8, 1.9)	0.212
IL-8, pg/mL	5.3 [4.7, 7.3]	8.1 [6.5, 13.2]	−2.2 (−3.5, −0.84)	0.001	−2.7 (−8.2, 2.8)	0.344
IL-17A, pg/mL	2.5 [1.0, 2.9]	2.9 [0.7, 3.8]	−0.44 (−1.4, 0.56)	0.395	−0.59 (−3.5, 2.3)	0.694
MCP-1, pg/mL	1.6 [−1.2, 2.8]	3.3 [1.6, 4.4]	−1.5 (−3.6, 0.56)	0.160	−1.4 (−4.8, 2.0)	0.415

Data are presented as median (25^th^ and 75^th^ percentiles)

*Abbreviations*: *B*, unstandardized regression coefficient; *CI*, confidence interval; *IL*, interleukin; *TNFα*, tumor necrosis factor-alpha; *hs-CRP*, high-sensitivity C-reactive protein; *PAI-1*, plasminogen activator inhibitor-1; *8-OHdG*, 8-hydroxy-2-deoxyguanosine; *MCP-1*, monocyte chemoattractant protein-1

†Unadjusted quantile regression

‡ Quantile regression adjusted with waist-to-hip ratio, education level, knowledge of indoor air pollution, use of air purifier, smoking of the family members in the house, burning incense at home, total duration of exercise, fine particulate matter of 2.5 or less in diameter, and nitrogen dioxide

## Discussion

This study examined the inflammatory responses of temple workers and volunteers to air pollutants from burning incense. The exposed group showed higher inflammatory levels than the control group in both hot and cold weather seasons, and environmental incense emission was associated with changes in serum biomarkers. In this study, the control group participants had higher PM_2.5_ and NO_2_ concentrations compared to the exposed group. However, the inflammatory levels in the control group were lower than those in the exposed group. This discrepancy might be due to the fact that the PM_2.5_ and NO_2_ concentrations were based on official data from the TAQMN near their homes, rather than their actual PM_2.5_ exposure data. Although the representation of exposure situations in the exposed group during incense burning at the temple or at home is limited, the inflammatory levels still reflect their true levels of air pollution exposure. The exposed (temple) group experienced long-term exposure to higher concentrations of indoor air pollutants both at the temple and at home. Our ongoing investigation (unpublished) has revealed that PM_2.5_ concentrations at the temple exceed Taiwan’s standard by 5–18 times. Additionally, participants in the exposed group, following their religious practices, burn incense at home frequently, leading to high concentrations of PM at home (Lung et al. 2021). Indoor burning of incense and joss papers results in PM_2.5_ and PM_1_ concentrations of 277.3 μg/m^3^ and 201.0 μg/m^3^, respectively (Lung et al. 2021). Moreover, PM_2.5_ from incense emissions indoors is approximately 1.53 ± 1.79 times higher than outdoor concentrations when burning incense for 30 to 60 min (Lung et al. 2021; Hien et al. 2022). Another study in Taiwan demonstrated that PM_10_ and PM_2.5_ concentrations were highest within 30 min after incense burning (Guo et al. 2020a, b, c). Consequently, the exposed group may have high exposure to air pollutants both at the temples and at home, potentially impacting their inflammatory biomarker reaction for long period compared to the control group.

Compared with the control group, the exposed group exhibited lower scores in their knowledge of air pollution and higher frequency of incense burning, which may have resulted in exposure to a higher concentration of indoor air pollutants; moreover, this group did not apply air pollution prevention interventions. The temple usually holds about 13 important festivals in the cold weather season, during which many worshippers visit the temple and burn incense, increasing the health risks of temple workers and volunteers who stay in the temple for prolonged periods. We found that the cold season exhibited a peak in the frequency of temple religious activities and festivals and the number of worshippers. Lung symptoms, mMRC scores, and FEV_1_ were significantly different between the two groups in the cold season; thus, in addition to atmospheric factors, intensive religious festivals during the cold season could influence the air quality of the temple and increase the health risk of temple workers and volunteers. Temple workers and volunteers who have long-term exposure to PM from incense burning are at risk of respiratory disease, various infectious diseases, inflammatory and autoimmune disorders, and cancer.

In addition to ambient particles, indoor incense burning releases synergistic PM and PM components that deteriorate health (Lin et al. 2008; Chen et al. 2017; Tse et al. 2011) and elicit an inflammatory response (Niu et al. 2019; Lin et al. 2012; Aalapati et al. 2014; Zhao et al. 2019). A previous study found that the PAH levels inside a temple were much higher than those in the outside environment and that temples were significant PAH and TSP producers, thus contributing to serious health hazards in local workers (Yadav et al. 2022). However, in the current study, 8-OHdG levels did not significantly differ between the two groups. Indoor air pollutants may differ in the groups, but we did not control for the involvement of participants in specific occupations and did not monitor PAH levels. A future examination of PAH levels and biomarkers between the two groups may reveal notable findings.

Incense burning generates heavy PM_2.5_ and NO_2_ emissions, which can induce oxidative stress (Niu et al. 2019) and stimulate an increase in inflammatory cytokines, including IL-1, IL-6, IL-8, IL-17A, and TNF-α (Lin et al. 2012; Aalapati et al. 2014; Zhao et al. 2019). In our study, temple workers and volunteers with long-term exposure to incense emissions had significantly higher levels of inflammatory responses than the control group. On examining the biomarkers from the serum of the exposed group, we found that TNF-α and IL-1*β* showed significantly higher levels, thereby releasing high levels of other inflammatory cytokines, such as IL-17, which could initiate the infiltration of neutrophils into the airways by inducing the release of IL-8 (Zou et al. 2017). Hence, the condition of cytokines in our study might reflect an early inflammation stage (see Table 3).

In the univariate analysis, serum IL-6 and IL-8 levels were significantly higher in the exposed group, consistent with findings from other studies. For example, Lin et al. (2012) found that the exposure of human coronary artery endothelial cells (HCAECs) to particles and PAHs caused an increase in IL-6. IL-6 production is often associated with increased levels of particles (Hassanvand et al. 2017; Ramos et al. 2017). Another study reported that short-term exposure to NO_2_ increased IL-8 levels (Dadvand et al. 2014), which could explain why the control group had significantly higher levels of EBC IL-8 with higher exposure to NO_2_ in the cold season.

Although the expression of inflammatory cytokines was demonstrated using the univariate analysis, after controlling for confounding variables (such as waist-to-hip ratio, education level, knowledge of indoor air pollution, use of air purifiers, smoking by family members in the house, exposure to incense burning, total duration of exercise, and PM_2.5_ and NO_2_ levels), only IL-1*β*, IL-17A, and PAI-1 showed significant differences; there were no differences in EBC. The PAI-1 in our analysis was significant, which was consistent with the findings of Wang et al., who reported that short-term exposure to air pollutants, including PM_2.5_ and NO_2_, is positively associated with PAI-1 (Wang et al. 2022). Green et al. (2016) reported that PAI-1 levels were related to long-term exposure to PM_2.5_ in middle-aged women. The long-term effects of incense burning on inflammation in the blood require further investigation. Although the preceding discussion mainly focused on the impact of air pollution on inflammation, it is essential to explore whether incense burning could elicit a similar response in future research.

The results of the current study showed that EBC did not differ after controlling for confounding variables. It could be possible that the inflammatory cytokines in EBC showed no obvious changes for participants in exposing to air pollutant for a long time, and the results of EBC reactions of our study were consistent with those of the study by Hu et al., who observed the effects of long-term exposure to traffic-related air pollution and found that EBC IL-6 is not associated with the distance of road and pollution exposure (Hu et al. 2016). Nevertheless, a previous study reported that the levels of EBC IL-6 and IL-8 increased on the third day after exposure, and TNF-α and IL-17 levels in EBC increased in the first 4 days after exposure to PM_2.5_; particularly, on the first day, the effects of pollutants on the levels of these cytokines in EBC were remarkable. Moreover, when comparing the effect of the 4-day moving average with the 7-day moving average of PM_2.5_, it was observed that higher levels of TNF-α and IL-17 in EBC may suggest inflammation occurring in the air passages and subsequently in the lungs (Alves et al. 2018). Another study found a 38.73% increase in EBC TNF-α with exposure to PM_2.5_ in the past 24 h (lag 0), but a decrease at lag 4 and lag 5. Additionally, a regional difference was observed in the changes of IL-6 in EBC with exposure to PM_2.5_, and a strong negative correlation between IL-6 and PM2.5 was observed at lags 0, 3, and 4. However, EBC IL-6 was not significantly related to PM_2.5_ in a less polluted group (Sabeti et al. 2021). These findings suggest that EBC could potentially reflect short-term inflammatory reactions, and emphasize the importance of designing EBC examinations for specific events to better understand the systematic expression of cytokines in EBC.

Table 2 shows lower biomarker results in the cold season compared to the warm season, which is unexpected based on previous studies. This discrepancy might be due to the influence of the COVID-19 pandemic. During the pandemic, lockdowns in Chinese cities led to reduced air pollution levels in Taiwan during the cold season (Griffith et al., 2020). In contrast, in the warm season of 2020, the Taiwanese government eased restrictions on tourism, resulting in increased pollution as people opted for self-driving. Additionally, mask-wearing during the pandemic might have affected the detection of biomarker differences caused by PM_2.5_ exposure. Nonetheless, this study emphasizes the impact of incense burning. Therefore, the most crucial aspect is the comparison between the exposed and control groups within the same season (Table 3), where biomarkers in the exposed group were observed to be higher than those in the control group

Ambient particles within the temple contain not only PM but also various toxic chemicals, such as PAHs, and these carcinogens probably cause a discrepancy in the mechanism of inflammatory cytokines that affect the health of temple workers and volunteers. This aspect needs to be addressed in future studies.

### Limitations

Our study had some limitations. First, our study only included 39 temple workers and volunteers, as well as 28 residents, which did not meet our planned sample size (*n*=128) due to the impact of the COVID-19 pandemic. This limitation has resulted in underpowered results for our study. To obtain more precise findings, larger-scale studies with an adequate number of participants are crucial. Additionally, the indoor PM levels varied based on participants’ characteristics and behaviors, such as exposure to smoking and incense burning. We relied on official monitoring stations for PM concentrations, which might not accurately represent individual PM exposure. Using personal monitoring equipment would provide more precise concentration estimations. Moreover, EBC collection was not conducted during specific events or worship dates, limiting the observation of short-term inflammatory reactions. To address this, future studies should design EBC examinations during particular events or dates to observe such changes in inflammatory cytokines.

## Conclusion

In conclusion, adapting the TAQMN data cannot specifically estimate the effects of indoor pollutants on inflammation in individuals, but our analyses of the study results accorded with our study hypothesis. Individual PM monitoring remains the first-priority method for future studies. Although the current findings hardly predict the endpoint of inflammation in causing disease or acute deterioration, our results showed that burning incense may significantly affect inflammatory responses and clinical symptoms. Additionally, our findings provide a guide for selecting a potential biomarker as an indicator to assess clinical diseases for further preventive health care and education.

Burning incense for worship is an age-old practice; and thus, an intervention to protect temple workers’ health is complex. There is evidence that some incense brands produce lower toxicity from combustion. Working with manufacturers, in addition to educating the public, may be an important first step in protecting the health of those exposed to burning incense. Implementing policies that promote adequate ventilation, limit exposure time and burned amount, and raise awareness about incense burning risks and how to minimize exposure may mitigate associated health risks. In some cases, alternative methods of aromatherapy, such as diffusers or essential oils, which do not produce smoke or harmful particles, may represent an acceptable and safer option.

## Data Availability

The data that have been used is confidential.
